# The effect of depression status change on daily cigarette smoking amount according to sex: an eleven-year follow up study of the Korea Welfare Panel Study

**DOI:** 10.1186/s12889-021-11362-y

**Published:** 2021-07-03

**Authors:** Soo Hyun Kang, Wonjeong Jeong, Sung-In Jang, Eun-Cheol Park

**Affiliations:** 1 Department of Public Health, Graduate School, Yonsei University, Seoul, Republic of Korea; 2 Institute of Health Services Research, Yonsei University, Seoul, Republic of Korea; 3 Department of Preventive Medicine, Yonsei University College of Medicine, 50 Yonsei-ro, Seodaemun-gu, Seoul, 03722 Republic of Korea

**Keywords:** Depression, Daily cigarette amount, Smoking cessation, Smoking

## Abstract

**Background:**

In the past decade, the Korean smoking rate has only decreased by 3%, despite several smoking control policies. There is a need for such policies to take smokers’ psychological characteristics into account. Depression is a well-known contributor to failed smoking cessation. This study aimed to examine the effect of smokers’ depression status changes on their daily cigarette smoking amount (DCA).

**Methods:**

This study used a sample drawn from the Korea Welfare Panel Study (KoWePS) waves 3 (2008) to 13 (2018). The DCA refers to the number of the cigarettes smoked per day at the time of the survey. Depression was measured using an 11-item version of the Center for Epidemiologic Studies Depression Scale (CESD-11). A generalized estimating equation (GEE) model was employed to analyse the effect of change of depression status on DCA.

**Results:**

The 2008 baseline included a total of 1821 participants: 1645 males and 176 females. The Yes→No male depression status group had lower DCA (β = − 0.631, *p*-value = 0.0248) than the No→No group. The Yes→No male depression status group that began smoking before age 19 had lower DCA (β = − 0.881, *p*-value: 0.0089) than the No→No group that started smoking before 19.

**Conclusions:**

We found that a change from depressed to non-depressed and non-depressed to depressed status is associated with decreasing and increasing DCA among men, respectively. Also, for smokers who began smoking before 19 years of age, the subgroup that went from depressed to non-depressed had much a lower DCA than general smokers. Thus, when treating people participating in smoking cessation programs, counsellors should check for depression symptoms and encourage individuals to pursue depression treatment simultaneously.

**Supplementary Information:**

The online version contains supplementary material available at 10.1186/s12889-021-11362-y.

## Introduction

In 2010, 8284 people died of either lung cancer or ischemic heart disease due to ongoing smoking [1]. The Korea National Health and Nutrition Examination Survey (KNHANES) calculated the national smoking rate as 22.3% in 2017, with 38.1% of men falling under the ‘current smoker’ category. Moreover, the past decade has only seen a 3% smoking decrease, down from 25.3%.

Since 1982, the Korean government has implemented various smoking control policies, such as regulating cigarette advertisements, adding warning labels to cigarette packs, restricting the sale of cigarettes to adolescents, banning smoking in public places and restaurants, and increasing cigarette prices [2]. Clearly, smoking control policies have not translated to a significant change in the national smoking prevalence. This indicates a need for research and policy development informed by both the sociodemographic and psychological characteristics of smokers.

To predict the success or failure of smoking cessation attempts, researchers have focused on the mitigating role of ‘major depressive disorder’ or ‘depressed mood’. A history of regular smoking was observed more frequently among those who experienced major depressive disorder at some point in their lives than it was among those who had not (non-depressed individuals) [3]. Some studies report that the history of major depressive disorder might imply the failure of smoking cessation [4, 5]. In addition, the relationship between depressed mood and past major depressive disorder has been supported in many studies on smokers [6, 7]. On the contrary, attempts to cease smoking, or successful smoking cessation may increase one’s risk of experiencing a major depression episode, irrespective of past major depressive disorder history [8, 9].

Self-reported daily cigarette amount (DCA) is an important factor used by many studies to assess the level of cigarette dependence. DCA has been used to determine what qualifies as ‘heavy’ dependence, or categorize those deemed ‘hardcore smokers’ [10–12]. Some studies have used DCA as a predictor for successful smoking cessation or of attempting cessation. Indeed, Hymowitz reported that individuals with lower daily cigarette consumption levels were statistically significant predictors of smoking cessation [13]. Similarly, Hyland found that individuals who reduced their smoking by more than 50% over 5 years were 1.7 times more likely to quit smoking in the 13 years after than those who did not [14].

Previous studies also demonstrate the relationship between depression and failure to quit smoking. However, the impact of smokers’ depression status change (e.g. from depressed status to non-depressed status) on their DCA has not been adequately assessed. Thus, this study sought to examine the effect of smokers’ depression status changes on their DCA.

## Methods

### Data and population

The study used data from the Korea Welfare Panel Study (KoWePS), conducted by the Korea Institute for Health and Social Affairs and Seoul National University. The KoWePS is an annual longitudinal panel survey that was undertaken from 2006 to 2018. This data is suitable for low-income policies or poverty studies because about 50% of samples were low income earners with a median income of 60% or less. This study used a sample drawn from waves 3 (2008) to 13 (2018). The initial 2008 baseline data included 16,613 individuals from 6314 households.

To analyze the effect of smokers’ depression status change, we only included currently smoking individuals who are 19 years old and older (excluded *n* = 13,591 in 2008). From there, we further excluded 1201 individuals with missing data for used variables in this study. Thus, the 2008 data included a total of 1821 individuals: 1645 males and 176 females.

### Measures

#### Daily cigarette smoking amount (DCA)

The outcome variable is Daily cigarette smoking amounts, referring to the number of the cigarettes smoked per day at the time of the survey.

#### Change in depression status

The main independent variable is change in depression status, which was measured by the 11-item version of the Center for Epidemiologic Studies Depression Scale (CESD-11). The CESD-11 is a shorter version of the original 20-item version, and is a self-reported screening tool which is well validated [15]. The total score of the CESD-11 was calculated by adding the scores for all 11 questions and multiplying these values by 20/11 to meet the total sum of the original form. Scores of 16 and higher were considered evidence of likely depressed status [15–17].

Depression status changing was measured as change in depression status in a previous year and the subsequent year. We categorised them into four groups: No→No (persistence of no depression status), No→Yes (newly being in depression status), Yes→No (exiting from depression status), and Yes→Yes (persistence of depression status).

### Covariates

Demographic, socioeconomic and health-related factors were included (S.Table 1). Demographic variables included sex, age, and region. Socioeconomic variables included education level, marital status, and income level (quartile). The sum of income reported by the household members was assigned to all of them as the household income. In the present study, we divided individuals into quartile using their equivalized household income, which considers the square root of the number of household members. Health-related factors included age at smoking commencement, alcohol consumption, and the absence or presence of chronic disease.

### Statistical analysis

We calculated the distribution of the general characteristics at baseline. An analysis of variance (ANOVA) test was used to analyze the mean DCA at the first time point, for change of depression status categories. To analyze the effect of change of depression status on DCA, we used the generalized estimating equation (GEE) model. The GEE model is a more efficient and unbiased regression estimate for analysing longitudinal or repeated measures in research designs with non-normal response variables [16]. We evaluated whether the DCA in each subsequent year was increased after depression status transitions over two consecutive years. Statistical analyses were performed using the GENMOD procedure in SAS version 9.4 (SAS Institute, Inc., Cary, NC, USA). The results were considered statically significant if the *p*-value was less than 0.05.

## Results

Table 1 presents the general characteristics at the first change time point (2008 → 2009). Of the 1821 participants, 86.9% of males and 64.2% of females were in the No→No group. The Yes→Yes group was the smallest, accounting for 2.7% of males and 10.2% of females. Among males, the No→Yes group had the highest mean DCA (18.6, SD: 9.88), and the Yes→No group had the smallest (16.40, SD: 6.29). Among females, the Yes→No group had the highest mean (13.95, SD: 8.48) DCA, and the No→No had the smallest (10.54, SD: 6.15). The mean ages at smoking initiation were 20.20 (SD: 4.44) in male and 32.54 (SD: 11.61) in female.

**Table 1:** General characteristics at the first change time point (2008 → 2009)

Variables	Daily cigarette smoking amount (DCA)											
	Male					***P*** value	Female					***P*** value
	N	%	MEANS	±	SD		N	%	MEANS	±	SD	
**TOTAL**	**1645**	**100**	**17.74**	**±**	**8.30**		**176**	**100**	**11.32**	**±**	**6.72**	
**Change of depression status (2008 → 2009)**												
No → No	1429	86.9	17.80	±	8.34	0.2428	113	64.2	10.54	±	6.15	0.0764
No → Yes	82	5.0	18.60	±	9.88		26	14.8	11.15	±	6.73	
Yes → No	90	5.5	16.40	±	6.29		19	10.8	13.95	±	8.48	
Yes → Yes	44	2.7	16.82	±	7.30		18	10.2	13.67	±	7.44	
**Age at the smoking initiation (years)**												
Under than 19	529	32.2	19.11	±	8.82	<.0001	19	10.8	8.11	±	4.92	0.2523
19 or older	1116	67.8	17.09	±	7.96		157	89.2	11.71	±	6.82	
**Age (years)**												
19–29	150	9.1	15.03	±	6.31	<.0001	10	5.7	7.00	±	2.87	0.0671
30–39	421	25.6	17.71	±	7.84		10	5.7	9.00	±	6.62	
40–49	383	23.3	19.77	±	8.45		24	13.6	11.46	±	6.21	
50–59	279	17.0	19.36	±	8.90		26	14.8	12.88	±	7.94	
60–69	238	14.5	17.04	±	8.52		30	17.0	13.57	±	7.31	
≥ 70	174	10.6	14.01	±	7.24		76	43.2	10.72	±	6.27	
**Region**												
Metropolitan	669	40.7	17.39	±	7.92	0.0186	76	43.2	11.12	±	6.91	0.3972
Rural	976	59.3	17.97	±	8.55		100	56.8	11.47	±	6.61	
**Education level**												
Middle school or under	587	35.7	17.64	±	8.12	0.0172	134	76.1	11.51	±	6.24	0.4203
High school	846	51.4	18.17	±	8.54		33	18.8	11.18	±	6.91	
College or above	212	12.9	16.28	±	7.68		9	5.1	9.00	±	12.04	
**Marriage status**												
Living w spouse	1165	70.8	18.02	±	8.35	0.2954	40	22.7	10.08	±	5.44	0.0532
Living w/o spouse	480	29.2	17.04	±	8.15		136	77.3	11.68	±	7.03	
**Income level**												
Low	405	24.6	16.45	±	7.67	0.0238	70	39.8	11.50	±	6.52	0.4305
Lower middle	446	27.1	18.30	±	7.99		47	26.7	12.89	±	7.36	
Upper middle	428	26.0	17.81	±	8.11		38	21.6	10.03	±	6.06	
High	366	22.2	18.38	±	9.38		21	11.9	9.52	±	6.61	
**Alcohol consumption**												
Non-drinkers	311	18.9	17.77	±	7.61	0.0003	91	51.7	12.30	±	7.13	0.6671
Once a week or less	594	36.1	17.33	±	8.01		60	34.1	10.52	±	6.48	
2–3 times a week	408	24.8	17.03	±	7.97		14	8.0	9.93	±	4.92	
More than 4 times a week	332	20.2	19.31	±	9.57		11	6.3	9.36	±	5.95	
**Chronic diseases**												
No	976	59.3	17.98	±	8.24	0.5129	53	30.1	10.23	±	6.19	0.7133
Yes	669	40.7	17.38	±	8.39		123	69.9	11.79	±	6.91	

Table 2 indicated the results of the GEE model analysis of factors associated with DCA. The Yes→No male depression status group was the only one that showed a statistically significant decrease in DCA (β = − 0.631, *p*-value = 0.0248). Male participants who started smoking before 19 years old experienced greater DCA (β = 0.477, *p*-value = 0.0290) than those who began at 19 or later. Increased DCA was observed among male participants who were 30–59 years old (30–39: β = 1.728; 40–49: β = 2.280;50–59: β = 2.211), had a lower education level (middle school or under: β = 1.530; high school: β = 1.454), and consumed alcohol 2–3 times a week (β = 0.434). The low and low-middle income level group had decreased DCA (low: β = − 1.156; low-mid: β = − 0.518) compared to those with high income level. Among female participants, increased DCA was observed in individuals who were 50–59 years old (β = 3.164) and consumed alcohol 2–3 times a week (β = 1.516).

**Table 2:** The results of the generalized estimating equation analysis of factors associated with daily smoking amount

Variables	Daily cigarette smoking amount (DCA)					
	Male			Female		
	ß	S.E	***P*** value	ß	S.E	***P*** value
**Change of depression status**						
No → No	Ref.			Ref.		
No → Yes	0.414	0.338	0.2202	−0.134	0.458	0.7702
Yes → No	−0.631	0.281	0.0248	− 0.065	0.523	0.9006
Yes → Yes	−0.005	0.578	0.9933	−0.769	0.727	0.2904
**Age at the smoking initiation (years)**						
Under than 19	0.477	0.219	0.0290	−0.009	1.047	0.9934
19 or older	Ref.			Ref.		
**Age (years)**						
19–29	Ref.			Ref.		
30–39	1.728	0.302	<.0001	0.144	1.423	0.9196
40–49	2.280	0.344	<.0001	1.591	1.312	0.2253
50–59	2.211	0.396	<.0001	3.164	1.438	0.0278
60–69	0.880	0.472	0.0620	2.414	1.375	0.0792
≥ 70	−1.377	0.513	0.0073	2.207	1.458	0.1301
**Region**						
Metropolitan	Ref.			Ref.		
Rural	0.415	0.249	0.0956	−0.342	0.524	0.5136
**Education level**						
Middle school or under	1.530	0.448	0.0006	0.041	1.833	0.9822
High school	1.454	0.332	<.0001	0.827	1.652	0.6164
College or above	Ref.			Ref.		
**Marriage status**						
Living w spouse	Ref.			Ref.		
Living w/o spouse	−0.126	0.243	0.6025	0.115	0.514	0.8228
**Income level**						
Low	−1.156	0.223	<.0001	−1.167	0.599	0.0514
Lower middle	−0.518	0.194	0.0075	−0.314	0.601	0.6008
Upper middle	−0.249	0.172	0.1483	−0.418	0.536	0.4348
High	Ref.			Ref.		
**Alcohol consumption**						
Non-drinkers	Ref.			Ref.		
Once a week or less	−0.089	0.197	0.6515	0.371	0.468	0.4286
2–3 times a week	0.434	0.220	0.0485	1.516	0.568	0.0076
More than 4 times a week	1.972	0.252	<.0001	2.080	0.676	0.0021
**Chronic diseases**						
No	Ref.			Ref.		
Yes	−0.272	0.142	0.0547	−0.200	0.352	0.5696
**Year**						
2009	Ref.			Ref.		
2010	−0.243	0.186	0.1915	−0.238	0.495	0.6303
2011	−0.454	0.196	0.0206	−0.195	0.512	0.7029
2012	−1.389	0.255	<.0001	−0.399	0.986	0.6854
2013	−1.552	0.269	<.0001	−0.779	1.080	0.4706
2014	−1.779	0.278	<.0001	−1.039	1.011	0.3042
2015	−2.991	0.287	<.0001	−1.696	1.073	0.1137
2016	−2.908	0.281	<.0001	−1.865	1.097	0.0889
2017	−2.870	0.288	<.0001	−1.920	1.076	0.0743
2018	−2.795	0.301	<.0001	−1.252	1.067	0.2405

Table 3 outlines the analysis, gradually narrowing subjects down to those with ≥20 DCA. Among those with ≥10 DCA, male in the Yes→No group had lower DCA (β = − 0.556) than those in the No→No group, which is similar to Table 2. Among those with ≥15 DCA, males in the No→Yes group had higher DCA (β = 0.789) than the No→No group. Analogously, males in the No→Yes category had higher DCA (β = 0.997) than the No→No group among participants with a ≥ 20 DCA. Among these, only the results for ≥20 DCA was statistically significant.

**Table 3:** The results of the generalized estimating equation analysis among smokers with more than 10 or 20 daily smoking amount

Change of depression status	Daily cigarette smoking amount (DCA)					
	Male			Female		
	ß*	S.E	***P*** value	ß*	S.E	***P*** value
**Among smokers with ≥ 10 daily amount**						
No → No	Ref.			Ref.		
No → Yes	0.453	0.363	0.2119	0.005	0.491	0.9914
Yes → No	−0.556	0.300	0.0637	0.031	0.572	0.9570
Yes → Yes	0.768	0.619	0.2144	−0.712	0.681	0.2963
**Among smokers with ≥ 15 daily amount**						
No → No	Ref.			Ref.		
No → Yes	0.789	0.407	0.0526	−0.133	0.512	0.7953
Yes → No	−0.280	0.295	0.3427	0.869	0.578	0.1327
Yes → Yes	0.295	0.654	0.6520	0.854	0.669	0.2015
**Among smokers with ≥ 20 daily amount**						
No → No	Ref.			Ref.		
No → Yes	0.997	0.416	0.0164	−0.463	0.455	0.3090
Yes → No	−0.360	0.301	0.2314	0.237	0.591	0.6889
Yes → Yes	0.335	0.667	0.6158	0.876	0.654	0.1805

* Age, region, marital status, educational level, income level, chronic disease, alcohol consumption, and survey year were adjusted

Table 4 outlines the subgroup analysis regarding the effects of change of depression status on DCA, according to age at the smoking initiation. The male Yes→No group who initiated smoking before 19 had lower DCA (β = − 0.881) than the male No→No group who initiated smoking before 19. Similarly, the Yes→No male group members living with spouses had lower DCA (β = − 1.088) than the No→No ones, although these are only statistically significant result in this analysis.

**Table 4:** Subgroup analysis of daily smoking amount stratified by age at the smoking initiation

Variables	Daily cigarette smoking amount (DCA)										
	No → No		No → Yes			Yes → No			Yes → Yes		
	ß*		ß*	S. E	***P*** value	ß*	S. E	***P*** value	ß*	S. E	***P*** value
**Male**											
**Age at the smoking initiation (years)**											
Under than 19	Ref.		0.163	0.395	0.6799	−0.881	0.337	0.0089	− 0.357	0.710	0.6151
19 or older	Ref.		0.166	0.702	0.8132	−0.111	0.519	0.8301	−0.022	0.966	0.9815
**Female**											
**Age at the smoking initiation (years)**											
Under than 19	Ref.		−0.245	0.508	0.6294	0.196	0.664	0.7677	−0.458	0.709	0.5183
19 or older	Ref.		0.756	0.810	0.3503	0.131	0.862	0.8795	−0.107	0.979	0.9126

* Age at the smoking initiation, age, region, marital status, educational level, income level, chronic disease, alcohol consumption, and survey year were adjusted

## Discussion

This study examined the effect of smokers’ depression status change on their daily cigarette smoking amount (DCA). Findings suggest that emerging from a depressed status is related to a decreased DCA among males. For males who smoke more than 20 cigarettes per day, depression status appears to increase DCA. Among males who began smoking before age 19, and males living with spouses, Yes→No depression status groups had a lower DCA than the No→No groups.

Smokers experiencing significant depression are more likely to begin smoking, due to the antidepressant effect of nicotine. In addition, nicotine boosts bioavailability of serotonin, whose mechanism is similar to some antidepressants [18]. Another possible explanation about the relationship between changing depression status and smoking is individuals’ socioeconomic status. It is clear that low socioeconomic status results in depression. Poverty and earning below the poverty threshold is associated with depression [19]. Also, there is a strong link between poverty and smoking. Individuals exiting poverty are more likely to quit smoking, and individuals whose living were below the poverty threshold is more likely to continue smoking [20, 21]. Not only the individual level socioeconomic status but also the neighbourhood poverty which is area level socioeconomic status was a risk factor for increased smoking prevalence among 30–39 aged adult participants in Seattle [22]. The association of socioeconomic status with depression status and consistent smoking may be associated with our finding. Hence, depression contributes to increased DCA. Moreover, depression encourages smokers, particularly those who smoke more than 20 cigarettes per a day, to persist in this behaviour despite the health risk. It is, therefore, necessary to help smokers seek alternative, healthier antidepressant resources, or assistance to weather a financial crisis, or to encourage smokers with depressive symptoms to consult mental problem professionals.

Much like smoking from an early age was associated with increased DCA, several studies reported the association between the age of smoking initiation and smoking cessation as well as strong nicotine dependence [23–25]. Indeed, a study focused on smokers who visited the Center for Cancer Prevention and Early Detection (in Korea’s National Cancer Center) reported that nicotine dependence was much higher among those who started smoking aged 19 or earlier (compared to those who started aged 25 or later) [26]. Of significance in our study is the fact that male smokers who started before 19 years of age had a significantly increased DCA than those started later. Also, smokers who began before 19 years of age and changed from depressed to non-depressed status had a much lower DCA than general smokers with the same change pattern. This may provide evidentiary support for the implementation of smoking cessation programs by detecting depression symptoms and encourage to participating in antidepressant programs in parallel with smoking cessation programs for smokers with early initiation age.

This study demonstrated no statistically significant results among females. This is likely related to the low self-reporting rate among female smokers in Korea. This small number of female smokers in this study data might be underestimated by self-reported responses. The notion of an underestimation issue in this demographic is supported by the Korea National Health and Nutrition Examination Survey (KNHANES) study. By comparing the number of females who self-reported as smokers with the number confirmed to be through urine cotinine examination, a significant discrepancy between the prevalence of self-reported and biochemically-verified female smokers [27]. To identify the effect depression status change on female smokers, further study is needed, using data obtained from other smoking detection methods, e.g. urine cotinine examination.

This study has several limitations. First, since the KoWePS largely represents low income households, study findings are somewhat limited when it comes to generalizing to the national population, and high income households in particular. Second, because of data limitations, the analysis could not include clinical laboratory results (such as those from urinary cotinine level exams) that show the degree of smoking intensity. Third, the use of self-reported responses means that measurement issues such as reporting bias and recall bias may have confounded the findings.

Despite the limitations, the present study has several strengths. First, we used data from a nationwide survey with randomly sampled longitudinal data and a 10-year follow up. As the KoWePS largely represents low income households, it can be generalized to South Korea’s low-income population. Second, previous studies have examined the association between depression and smoking cessation success. However, this study used a lagged GEE model to determine the ability of depression status change (which may occur through treatment) to lessen daily smoking cigarettes among smokers.

## Conclusions

In conclusion, this study examined the effect depression status change on smokers’ DCA. We found that change from depressed to non-depressed and non-depressed to depressed status is associated with decreasing and increasing DCA, respectively. Among those who began smoking before age 19, the subgroup that changed from depressed to non-depressed status had a much lower DCA than general smokers with the same change pattern. These findings may support the incorporation of depressive symptom treatment into smoking cessation programs.

## Supplementary Information

**Additional file 1.**

## Data Availability

The KoWePS data supporting this article is openly published and provided for the research study by the Korean government. The data is openly available in https://www.koweps.re.kr:442/main.do by submitting oath and data utilization plan.

## Abbreviations

DCA: Daily cigarette smoking amount
KoWePS: Korea Welfare Panel Study
CESD-11: An 11-item version of the center for epidemiologic studies depression scale
ANOVA: Analysis of variance
GEE: Generalized estimating equation
KNHANES: Korea National Health and Nutrition Examination Survey
SD: Standard deviation
SE: Standard error
