# Supplementary material for: The effect of depression status change on daily cigarette smoking amount according to sex: an eleven-year follow up study of the Korea Welfare Panel Study
Source: BMC Public Health. 2021 Jul 3;21:1304. doi: 10.1186/s12889-021-11362-y (PMC8254959; doi:10.1186/s12889-021-11362-y)
Supplement: Supplementary file 1 — Additional file 1. [file 12889_2021_11362_MOESM1_ESM.docx]

| Study variables | Description and categories |
| --- | --- |
| Change of depression status | Change in depression status (yes=having CESD-11 scores of 16 and higher; no=having CESD-11 scores lower than 16) in a previous year and the subsequent year (1=persistence of no depression status; 2=newly being in depression status; 3=exiting from depression status; 4=persistence of depression status) |
| Age at the smoking initiation | Age when individuals commenced smoking at first (1=before age of 19; 2=at age of 19 or after) |
| Age | Individual’s age in each year (1=19-29 years; 2=30-39 years; 3=40-49 years; 4=50-59 years; 5=60-69 years; 6=≥70 years) |
| Region | Region of residence in each year (1=metropolitan; 2=rural) |
| Education level | Education level of individuals (1= middle school or under; 2=high school; 3=college or above) |
| Marriage status | Individual’s marital status in each year (0=unmarried/divorced/bereaved/separated; 1=married and living with spouse) |
| Income level | Total income of all the household members quartile in each year (1=low; 2=lower middle; 3=upper middle; 4=high) |
| Alcohol consumption | Frequency of alcohol consumption in the recent year (0=never; 1=once a week or less; 2=2-3 times a week; 3=more than 4 times a week) |
| Chronic disease | Diagnosis of any chronic disease in each year (0=absence; 1=presence) |
| Year | Subsequent year in each two consecutive years (1=2009; 2=2010; 3=2011; 4=2012;5=2013;6=2014;7=2015;8=2016;9=2017;10=2018) |

S.Table 1. Explanatory variables
